# Characterization and dynamics of pericentromere-associated domains in mice

**DOI:** 10.1101/gr.186643.114

**Published:** 2015-07

**Authors:** Patrick J. Wijchers, Geert Geeven, Michael Eyres, Atze J. Bergsma, Mark Janssen, Marjon Verstegen, Yun Zhu, Yori Schell, Carlo Vermeulen, Elzo de Wit, Wouter de Laat

**Affiliations:** Hubrecht Institute-KNAW & University Medical Center Utrecht, 3584 CT Utrecht, The Netherlands

## Abstract

Despite recent progress in genome topology knowledge, the role of repeats, which make up the majority of mammalian genomes, remains elusive. Satellite repeats are highly abundant sequences that cluster around centromeres, attract pericentromeric heterochromatin, and aggregate into nuclear chromocenters. These nuclear landmark structures are assumed to form a repressive compartment in the nucleus to which genes are recruited for silencing. We have designed a strategy for genome-wide identification of pericentromere-associated domains (PADs) in different mouse cell types. The ∼1000 PADs and non-PADs have similar chromatin states in embryonic stem cells, but during lineage commitment, chromocenters progressively associate with constitutively inactive genomic regions at the nuclear periphery. This suggests that PADs are not actively recruited to chromocenters, but that chromocenters are themselves attracted to inactive chromatin compartments. However, we also found that experimentally induced proximity of an active locus to chromocenters was sufficient to cause gene repression. Collectively, our data suggest that rather than driving nuclear organization, pericentromeric satellite repeats mostly co-segregate with inactive genomic regions into nuclear compartments where they can contribute to stable maintenance of the repressed status of proximal chromosomal regions.

One of the major challenges in genome biology is to understand how the genome is organized and what factors control the spatiotemporal expression patterns of genes in different cell types. It is well established that higher-order organization of chromatin within the three-dimensional space of the nucleus is an important contributor to regulation of gene expression. In particular, long-range physical interactions of genomic elements in the nuclear space enable functional communication between genes and their regulatory DNA elements that can be hundreds of kilobases apart on the linear chromosome (for review, see de Laat and Duboule 2013). At the same time, genes must be protected from promiscuous influences of other regulatory elements and chromatin types, and active and inactive chromatin are kept apart in the nucleus (Bickmore and van Steensel 2013). How these tissue-specific topological configurations of chromatin are established and maintained in different cell types is still largely unknown.

Noncoding DNA is considered essential for the regulation of more complex spatiotemporal gene expression patterns in mammalian species (Gregory 2005; de Laat and Duboule 2013). Repetitive DNA sequences may account for more than two thirds of the mammalian genome (de Koning et al. 2011), yet their regulatory and architectural role remains largely enigmatic, partly because they are difficult to study with molecular biology techniques. Certain subclasses of DNA repeats have a propensity to come together to form visually recognizable structures in interphase nuclei. Pericentromeric satellite repeats from different chromosomes cluster together into nuclear landmark structures called chromocenters (Baccarini 1908), which in murine nuclei are easily visualized using fluorescent DNA dyes such as DAPI (4′,6-diamidino-2-phenylindole), due to their preference for megabase-long tandem arrays of A/T-dense “major” satellite repeats (Mayer et al. 2005; Probst and Almouzni 2008). This clustering appears to be cell type-specific, with large variations in the number, size, and radial position of chromocenters (Mayer et al. 2005; Solovei et al. 2009). Differentiation is generally accompanied by increased clustering into fewer but larger chromocenters and their relocation to the nuclear periphery (Beil et al. 2002; Weierich et al. 2003; Mayer et al. 2005; Wiblin et al. 2005). This dynamic clustering has a dramatic visible impact on the nucleus, suggesting that satellite repeats may be a driving influence on nuclear organization.

Pericentromeric satellite repeats are condensed into constitutive heterochromatin. This pericentromeric heterochromatin (PCH) is traditionally considered to comprise a repressive environment in the nucleus (Politz et al. 2013). Studies on position effect variegation (PEV) have shown that classic euchromatic genes become silenced in a proportion of cells when positioned in close linear proximity to (pericentromeric) heterochromatin as a result of chromosomal rearrangements or transgene integrations (Weiler and Wakimoto 1995). Initial studies using FISH to analyze nuclear localization of genetic loci in mouse lymphoid cells also reported cell-type-specific three-dimensional proximity to PCH in chromocenters only in cell types where these genes are not expressed (Brown et al. 1997, 1999). In human erythroid cells, the extensively studied beta globin gene relocates away from chromocenters upon gene activation (Francastel et al. 1999; Schubeler et al. 2000). However, many genes in hematopoietic and other cell types do not associate with chromocenters when transcriptionally inactive (Brown et al. 2001; Hewitt et al. 2004; Takizawa et al. 2008; Jost et al. 2011). Moreover, some genes have been shown to be transcribed even when associated with chromocenters (Lundgren et al. 2000; Sabbattini et al. 2001), and several genes that are embedded in heterochromatin appear to rely on a heterochromatic environment for expression in *Drosophila* (Wakimoto and Hearn 1990; Lu et al. 2000). Thus, from these studies on selected genes, the role of chromocenters in nuclear organization and gene expression is far from clear. To study the function of chromocenters in a systematic manner, we designed and applied a strategy for genome-wide identification of chromosomal regions frequently associated with pericentromeric satellite repeats in mouse cells.

## Results

### Systematic identification of genomic regions associated with pericentromeric satellite repeats

**Figure 1.**
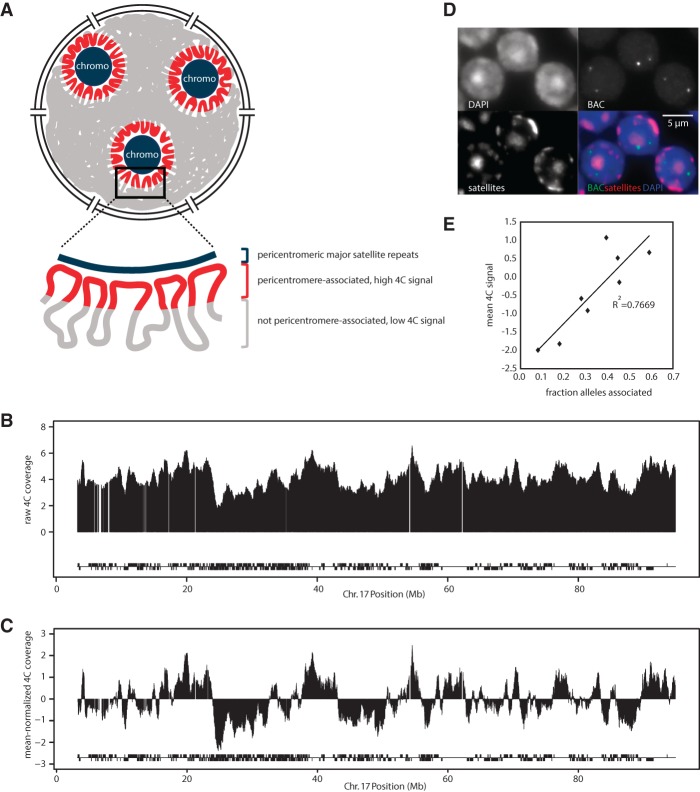
Identification of genomic regions associated with pericentromeric satellite repeats in chromocenters using “sat4C.” (*A*) Schematic view of pericentromeric satellite repeats in chromocenters (chromo) and their associated genomic regions in the nucleus. (*B*) Raw 4C coverage plot of sat4C profile of Chromosome 17 from mouse thymus. Blocks *below* sat4C map indicate gene positions on each strand. (*C*) Mean-normalized sat4C coverage profile. Same as in *B* but with the chromosomal mean subtracted to visually highlight regions with relatively high and low sat4C signals (see also Supplemental Fig. S1). Note that this subtraction is a visual aid, and values *above* or *below* the 0 line do not necessarily equal associated or nonassociated, respectively. (*D*) DNA FISH images showing probes used for sat4C validation in *E*. Scale bar, 5 μm. (*E*) Correlation between average sat4C signal and the frequency of pericentromeric association in thymus for eight chromosomal regions.

To map genomic regions associated with chromocenters in mouse (Fig. 1A), we used the 234-base pair (bp) major satellite repeat unit as a viewpoint in a chromosome conformation capture approach followed by high-throughput sequencing (4C-seq) (Simonis et al. 2006; Splinter et al. 2011). Pericentromeric DNA is organized in tandem arrays of ∼200,000 major satellite repeats (Martens et al. 2005), so we had to adapt the standard 4C strategy to prevent sequencing of the thousands of satellite-to-satellite ligation events (see Methods; Supplemental Fig. S1A). We applied this modified 4C procedure, referred to as “sat4C,” to primary adult mouse thymus tissue, an ENCODE-selected tissue for which many epigenetic data sets are available (The ENCODE Project Consortium 2011) that can assist in characterization of the associated regions. Sequencing reads from three biological replicates were mapped to a reduced mouse genome (Splinter et al. 2012) that contains only 4C fragment-end sequences. We computed coverage in running windows of 101 4C fragment ends across each chromosome (Fig. 1B) and subtracted the chromosome-wide average to visually highlight chromosomal regions with high and low 4C coverage, i.e., regions that are more or less frequently associated with pericentromeric satellites, respectively (Fig. 1C). These sat4C profiles were consistent across biological triplicate experiments (Supplemental Fig. S1B). To independently validate that sat4C profiles reflect high-order chromosome topologies, we performed DNA FISH to measure pericentromeric association frequency for eight chromosomal regions covering a broad range of sat4C signals. We found a high concordance between visual association frequency and sat4C signal (*r*^2^=0.7669 at distances < 0.3 μm) (Fig. 1D,E), demonstrating that our sat4C method reliably detects the association frequency of genomic intervals with chromocenters.

### Pericentromeric association segregates repressed chromatin from active chromatin

**Figure 2.**
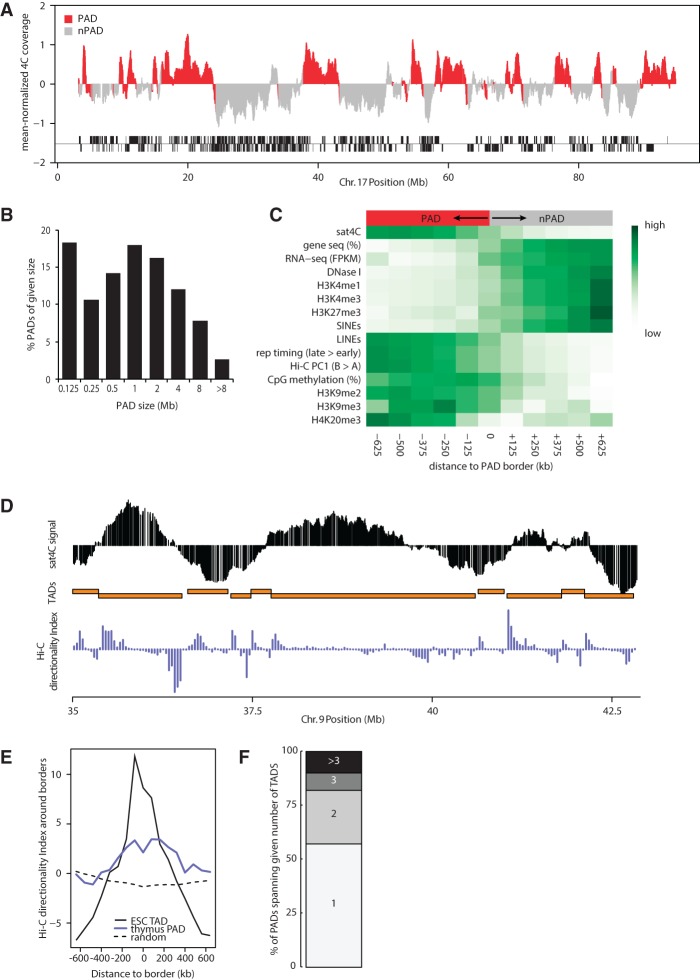
Pericentromeric association segregates repressed chromatin from active chromatin. (*A*) Sat4C profile of Chromosome 17 with designated PADs (red) and non-PADs (gray) based on a two-state hidden semi-Markov model (HSMM) on three biological replicate thymus samples. Blocks *below* the sat4C map indicate gene positions on each strand. Note that the HSMM is based on individual reads and may not perfectly align with the running-windowed sat4C signal (see also Fig. 1B). (*B*) Histogram plot with PAD sizes in thymus (median size 640 kb). (*C*) Heatmaps showing average enrichment scores for chromatin features in 125-kb windows around PAD borders. Color codes represent normalized values for each feature from minimum to maximum values. (*D*) Alignment of thymus sat4C profile with ESC Hi-C topological domains (TADs, orange blocks) and the ESC Hi-C directionality index (purple). (*E*) Enrichment of Hi-C directionality index (averaged over 80-kb windows) around PAD and TAD borders. Note that the absolute value of the index was taken to analyze the amplitude of the directionality bias. (*F*) Stacked column plot showing the percentage of PADs that span the number of neighboring TADs indicated.

Sat4C profiles displayed large continuous regions that preferentially associate with or locate away from chromocenters (Fig. 1B). To systematically characterize the associated regions, we applied a two-state hidden semi-Markov model (HSMM) to the reads mapped at individual fragment ends from biological replicates of three different mice to identify pericentromere-associated domains (PADs) (red in Fig. 2A) that alternate with non-PADs (gray in Fig. 2A). This analysis identified 845 PADs in the thymus that spread along the acrocentric chromosomal arms, varying in size from several kb up to almost 20 Mb (Fig. 2B), with a median size of 640 kb. This suggests that genes associate with chromocenters as part of larger genomic domains, rather than on a single gene basis, reminiscent of other domain-sized genomic features such as lamina-associated domains (LADs) (Peric-Hupkes et al. 2010) and topologically associating domains (TADs) (Dixon et al. 2012; Nora et al. 2012).

To investigate whether PAD distribution correlates with known epigenetic features of chromosomes, we aligned all PADs by their left and right borders and determined the average distribution of published genomic features (The ENCODE Project Consortium 2011) across these borders. This analysis revealed that chromatin composition differs dramatically on either side of PAD borders. PADs are relatively gene poor, deprived of short interspersed elements (SINEs), but enriched for long interspersed elements (LINEs) (Fig. 2C). The 5636 genes that do reside in PADs generally show low levels of expression. Consistent with low levels of expression, PADs are depleted of DNase I hypersensitive sites and other marks of active chromatin such as H3K4me1 and H3K4me3 and enriched for DNA methylation (Hon et al. 2013) and heterochromatin marks such as H3K9me2, K3K9me3, and H4K20me3 (the latter based on comparison with ChIP-seq data obtained from embryonic stem cell-derived terminal neurons [H3K9me2] [Lienert et al. 2011] and adult liver [H3K9me3 and H4K20me3] [Magklara et al. 2011], as no such data are available for thymus) (Fig. 2C). These signatures of pericentromere-associated regions are in agreement with previously published FISH and immunofluorescence data showing that chromocenters are surrounded by a halo of heterochromatic histone marks (Solovei et al. 2009; Eberhart et al. 2013). The gene repression in PADs is not mediated by Polycomb-group proteins, as the Polycomb-associated H3K27me3 mark is mostly associated with genes in non-PADs (Fig. 2C). Replication timing data from CD4^+^ single positive T lymphocytes (Weddington et al. 2008) suggested that the identified PADs replicate late in S phase (Fig. 2C), similar to pericentromeric satellite repeats themselves.

Genome-wide association maps of mammalian genomes have revealed that active and inactive regions are tightly segregated into an “A” and “B” compartment, with regions from either compartment preferentially associating with other regions from the same compartment (Lieberman-Aiden et al. 2009; Zhang et al. 2012). Consistent with late replication and enrichment of heterochromatic marks, PADs are associated with the closed and generally repressed B compartment (Fig. 2C). This prompted us to compare PADs to TADs, local chromatin interaction domains identified by Hi-C with differing chromatin signatures that are separated in the nucleus (Dixon et al. 2012; Nora et al. 2012). Since TADs are largely invariant between different cell types, we compared our thymus sat4C data to available Hi-C data from mouse ES cells (Dixon et al. 2012). Visual inspection of Hi-C matrices suggests that transitions in sat4C signal often coincide with TAD borders (Fig. 2D). To more systematically compare PAD and TAD borders, we analyzed the average amplitude of the Hi-C directionality index across their borders. This index, which quantifies the directional contact bias for genomic regions of 40 kb, typically peaks around the borders of topological domains (Dixon et al. 2012), showing a directional contact bias enrichment within ∼200 kb of aligned TAD borders (Fig. 2E). Similarly, albeit less pronounced, the amplitude of the directionality index was highest around PAD borders (Fig. 2E). This suggests that genomic regions preferentially associate with chromocenters as topological units, rather than as individual loci, which is consistent with PADs often spanning more than one neighboring TAD (Fig. 2F). Altogether, our data show that pericentromeric association in primary thymocytes tightly segregates large domains of repressed chromatin from active chromatin domains.

### Segregation of inactive chromatin around chromocenters is established during lineage commitment

**Figure 3.**
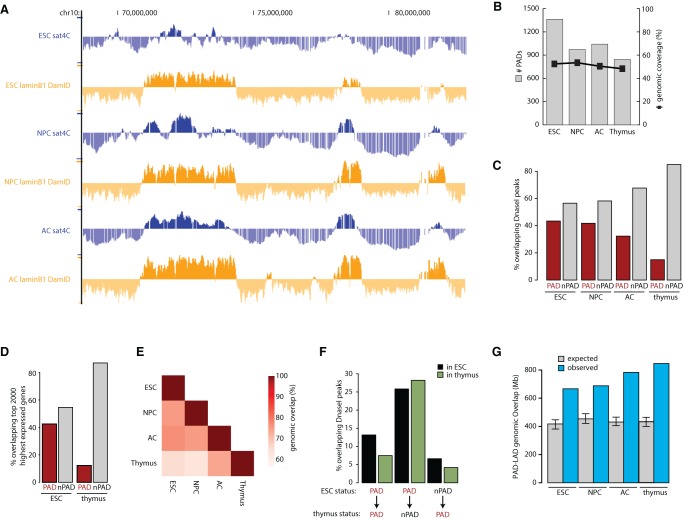
Segregation of inactive chromatin around chromocenters is established during lineage commitment. (*A*) Comparison of sat4C (blue) and Lamin B1 DamID (orange) profiles (taken from the UCSC Genome Browser) for the same genomic region in ESCs, NPCs, and ACs. (*B*) Histogram showing the number of PADs and the genomic coverage in each cell type. (*C*) Distribution of clusters of DNase I hypersensitive sites (hotspots) across PADs and non-PADs in each cell type. (*D*) Distribution of the top 2000 expressed genes across PADs and non-PADs in ESC and thymus. (*E*) Heatmap showing pairwise genomic PAD overlaps between all tissues examined as a percentage of PADs in the labeled tissues *below* the heatmap. (*F*) Comparison of DNase I hypersensitive sites in ESC and thymus for constitutive PADs, ESC-specific and thymus-specific PADs. (*G*) Histogram plot showing PAD-LAD overlap as genomic coverage (blue) and the 99% confidence intervals for expected overlap (gray with error bars) in each cell type based on randomization.

To follow PADs during lineage commitment, we next differentiated pluripotent mouse embryonic stem cells (ESCs) sequentially into in vitro–derived neural precursor cells (NPCs) and terminally differentiated astrocytes (ACs) (Peric-Hupkes et al. 2010). Sat4C profiles in these cell types revealed that PAD organization in ESCs appeared unusual in several aspects. The sat4C signal fluctuated more in ESCs than in more differentiated cell types (Fig. 3A), and more PADs were called in ESCs by our HSMM than in other cell types, while the total genomic coverage of PADs was not markedly different (Fig. 3B). Consistent with the latter, ESC PADs were generally smaller in size compared with other cell types (data not shown).

When investigating whether these physical differences were accompanied by any functional deviations, we found that in sharp contrast to thymus tissue (Fig. 2C), there were only minor differences in density of DNase I hypersensitive sites between PADs and non-PADs from ESCs (Fig. 3C). Sequential differentiation of ESCs into lineage-committed NPCs and ACs disclosed a progressive deviation between PADs and non-PADs in density of DNase I hypersensitive sites (Fig. 3C), with PADs clearly depleted in more differentiated cell types. Likewise, highly expressed genes are greatly enriched in non-PADs in differentiated thymus tissue but were almost as common in PADs as in non-PADs in ESCs (Fig. 3D). Other chromatin marks showed similar patterns (data not shown), suggesting that spatial assembly of inactive chromatin around the pericentromeric regions is largely absent in pluripotent ESCs and becomes gradually established as lineage commitment progresses.

### Chromocenters progressively contact already silenced genomic regions

We envisaged two explanations why inactive chromatin did not assemble around chromocenters in ESCs. First, PADs are transcriptionally active in ESCs and these same regions become repressed during differentiation. In this scenario, satellite association may serve as a bookmark for silencing later during differentiation. Alternatively, distinct inactive regions progressively replace active regions around chromocenters during differentiation. We found relatively limited overlap in PAD status between the four tissues analyzed (Fig. 3E), with 50.4% of the genome (51.5% of ESC PADs) sharing PAD or non-PAD status across all four tissues (data not shown). This suggests that PAD identity is often cell type-specific and that approximately half of the genome switches PAD status in one or more tissues.

To better understand this dynamic association, we distinguished constitutive PADs (regions consistently associated with satellites in all four cell types) from facultative PADs. Constitutive PADs appeared condensed in all tissues, as inferred from their low density in DNase I hypersensitive sites (Fig. 3F). In contrast, regions associated with chromocenters in ESCs (ESC PADs) that are no longer associated in the thymus (thymus non-PADs) already carried an open chromatin signature in ESC (Fig. 3F). Conversely, the newly associated regions in thymus that were still non-PADs in ESCs were already largely compacted in ESCs (Fig. 3F). So, instead of chromocenter-associated regions becoming progressively repressed, initially unbound repressed regions increasingly associate with chromocenters during differentiation. This explains why PADs gain an overall inactive chromatin signature during lineage commitment. It does not, however, reveal whether this is a consequence of inactive chromatin being recruited to chromocenters or, vice versa, chromocenters moving to inactive chromatin compartments.

### Chromocenters progressively overlap with inactive chromatin at the nuclear periphery

Inactive chromatin is preferentially located at the nuclear periphery (Deniaud and Bickmore 2009). Genome-wide profiling of LADs using Lamin B1 DamID (Guelen et al. 2008; Peric-Hupkes et al. 2010) has revealed the identity of these peripheral, predominantly inactive regions that together comprise almost 40% of the genome (Guelen et al. 2008; Peric-Hupkes et al. 2010). In contrast to PADs, LADs are highly conserved in ESCs, NPCs, and ACs (Peric-Hupkes et al. 2010). Thus, there appears to be no dramatic nuclear reorganization of the inactive compartment that preferentially locates to the nuclear periphery of most mammalian cell types.

Instead, microscopy studies have previously shown that chromocenters generally occupy more peripheral territory during lineage commitment (Weierich et al. 2003; Mayer et al. 2005; Wiblin et al. 2005). Consistently, we observed that PADs increasingly overlapped with the conserved LADs during lineage commitment (Fig. 3A,G). We consequently conclude that upon differentiation chromocenters localize more at the nuclear periphery, where they increasingly associate with locally accumulated inactive regions. Thus, more than actively recruiting chromosomal region segments for silencing, chromocenters migrate to an already existing nuclear compartment for association with inactive chromosomal regions.

### Induced proximity to chromocenters is sufficient for transcriptional repression

The repressed status of developmentally induced PADs prior to chromocenter association implies that pericentromeric satellite repeats are not responsible for silencing these regions. However, it does not preclude a role for chromocenters in active contribution gene repression. A hint that this may occur comes from the artificial recruitment of heterochromatin protein 1 variants (HP1) to a transgene, which was previously shown to cause increased association with chromocenters (Ayyanathan et al. 2003) and to induce gene silencing (Ayyanathan et al. 2003; Hathaway et al. 2012). However, these experiments did not allow discerning whether the two effects were causally related: Silencing could have been induced by pericentromeric recruitment, but pericentromeric association may also have been the consequence of HP1-induced gene silencing (Ayyanathan et al. 2003; Hathaway et al. 2012).

**Figure 4.**
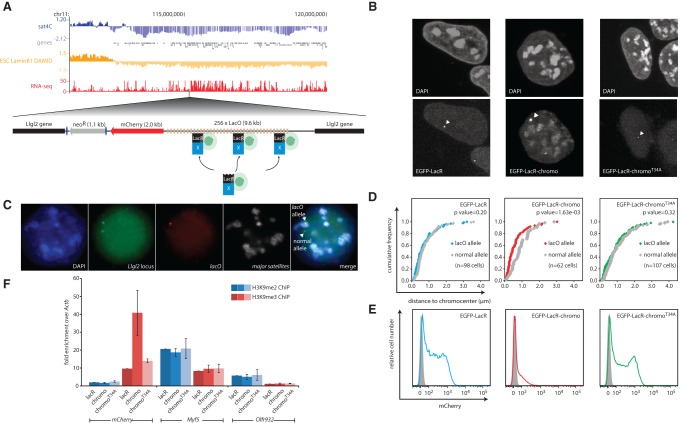
Induced proximity to chromocenters is sufficient for transcriptional repression. (*A*) Genomic view with sat4C, Lamin B1 DamID, and gene expression data for the *lacO* integration site. *Below* is the *lacO* transgene used in this assay. Note that EGFP-LacR fusion proteins are transiently transfected and recruited to the *lacO* array. (*B*) DAPI and EGFP distribution of the EGFP-LacR fusion constructs in mouse ESCs. Arrowheads highlight the position of the bright *lacO* integrated site. (*C*) Image series of three-color FISH strategy used to measure pericentromeric proximity of the *lacO* and untargeted allele in *D*. In this example, only the *Llgl2* allele (green) that overlaps with the *lacO* transgene (red) is associated with pericentromeric satellites (white). Images are maximum projections of a z-stack to simultaneously show both the normal and *lacO* allele. (*D*) Cumulative frequency plot of the distance of the *lacO* (color) and normal (gray) allele to the *nearest* chromocenter as measured by DNA FISH as shown in *C*. *P*-values are based on two-sample Kolmogorov-Smirnov tests. Numbers of cells analyzed (with one allele each) are indicated in parentheses. (*E*) FACS plots of mCherry expression levels. Gray peak represents auto-fluorescence in nontransgenic founder cells. (*F*) ChIP data showing enrichment values of H3K9me2 (blue) and H3K9me3 (red) levels relative to LacR transduced cells. Values represent averages and error bars the standard deviations from three independent ChIPs, normalized to the *Actb* promoter.

To distinguish between these possibilities, we used the bacterial lac operator-repressor (*lacO*/LacR) system (Robinett et al. 1996) in combination with two HP1 mutant proteins. First, we randomly integrated a DNA cassette carrying 256 *lacO* repeats with an adjoining *mCherry* reporter gene into the genome of mouse ES cells. We then selected a clone where the *lacO* cassette had landed in a large non-PAD/iLAD at the telomeric end of Chromosome 11 (Fig. 4A). We then recruited different LacR fusion proteins to this *lacO* platform: EGFP-LacR, EGFP-LacR-chromo, and EGFP-LacR-chromo^T34A^. The latter two fusion constructs only carry the chromodomain (chromo) of CBX1 (also known as HP1beta) that is responsible for binding to the pericentromerically enriched H3K9me3 mark (Bannister et al. 2001; Lachner et al. 2001; Nakayama et al. 2001) but lacks the hinge and chromoshadow domains that accommodate the protein-nucleic acid and most protein-protein interactions of CBX1 (Hiragami and Festenstein 2005). The threonine at position 34 in the chromodomain (position 51 in the CBX1 protein) is critical for H3K9me3 association, and binding to this heterochromatic mark is therefore disrupted in the chromo^T34A^ mutant (Ayoub et al. 2008). Concordantly, visual inspection showed that the fluorescently tagged chromo protein preferentially localized at DAPI-dense chromocenters. On the other hand, the chromo^T34A^ mutant showed a random nuclear distribution similar to LacR (Fig. 4B). Next, we determined whether these variants could recruit the *lacO* chromosomal binding sites to chromocenters. For this, we used three-color 3D FISH and labeled major satellites, the *lacO* allele, and the corresponding untargeted region on the Chromosome 11 homolog to simultaneously measure the distance of the untargeted and the *lacO* targeted allele to the nearest chromocenter (Fig. 4C,D). Binding of the chromodomain to the *lacO* platform led to increased association with chromocenters, while mutant chromo^T34A^ binding led to a similar distribution as the normal allele (Fig. 4D). This supports the idea that pericentromeric recruitment of the *lacO* locus is mediated by chromodomain-binding to chromocenter-accumulated H3K9me3 (Ayoub et al. 2008). Increased association with chromocenters was accompanied by robust silencing of the linked *mCherry* reporter gene, which was not observed when *lacO* was bound by chromo^T34A^ (and was not recruited to chromocenters) (Fig. 4E). The finding that a single amino acid substitution in the chromo domain not only abolishes locus recruitment to the chromocenters, as expected, but also fails to inactivate gene expression demonstrates that forced recruitment to chromocenters can be sufficient to induce transcriptional repression.

PADs are enriched for both the peripheral heterochromatin mark H3K9me2 and the classical chromocenter mark H3K9me3 (Fig. 2C). This contrasts with LADs, which are only enriched in the peripheral heterochromatin mark H3K9me2 (Guelen et al. 2008; Peric-Hupkes et al. 2010; Kind et al. 2013). To investigate which of these modifications is deposited upon recruitment to and silencing at chromocenters, we performed chromatin immunoprecipitation (ChIP) assays on the *mCherry* reporter gene. We found that the classical pericentromeric marker H3K9me3 (Bannister et al. 2001; Lachner et al. 2001), but not the peripheral H3K9me2 mark, was increased at the *mCherry* promoter upon induced pericentric recruitment (Fig. 4F). This increase was absent upon binding of the mutant chromodomain. Thus, induced recruitment to chromocenters leads to H3K9me3 deposition and gene repression. Collectively, our data suggest that the association of inactive chromatin with satellite sequences will often be the consequence of chromocenters preferably positioning themselves at already established inactive nuclear compartments, but that chromocenters can contribute to the repression of associated genes.

## Discussion

We present here the first systematic identification of genomic regions associated with the prevalent satellite repeat sequences that flank the centromeres of mouse chromosomes. The homogeneous tandem organization of major satellite repeats in mice enabled the use of a single 4C primer pair to simultaneously look from the tens of thousands of satellite repeats in each cell. We show by microscopy that the identified regions (PADs) have an increased probability to be close to chromocenters, the sites where pericentromeric repeat sequences spatially cluster. Thus, sat4C profiles predict the likelihood of association similar to other genomic contact maps (Kind et al. 2013), although a high sat4C signal does not necessarily imply that a region is associated to chromocenters in every cell. The probabilistic nature of higher-order genome organization, with cell-to-cell differences in numbers, shape, and nuclear location of chromocenters as well as in the genomic parts associated with them, limits the very quantitative interpretation of sat4C data. It compromises an exact definition of PAD boundaries where they exist, but sat4C profiles can very well be interpreted at the chromosomal domain level, as we have shown here. Chromocenter formation has also been observed in species such as fission yeast (Funabiki et al. 1993), plants (Fransz et al. 2002), and human (Manuelidis 1984; Bartholdi 1991; Alcobia et al. 2000; Weierich et al. 2003), and most hallmark pericentromere-associated proteins are highly conserved across these species. However, there is diversity in the sequence composition and length of pericentromeric repeats across species, and pericentromeric clustering is not always as extensive as in mouse nuclei. Given this diversity, the strategy presented here is only applicable to mouse satellite sequences. It therefore remains to be seen whether our findings in mice also apply to human and other species.

### Association between inactive chromatin and chromocenters is acquired during lineage commitment

Our data show that the preferred assembly of inactive chromatin around chromocenters is an acquired feature of differentiated cells; it is not, or barely, appreciable in ESCs. The undefined chromatin signature of PADs in ESCs fits well with previous observations that inactive chromatin is unusually disorganized in pluripotent stem cells (de Wit et al. 2013) and that chromocenters appear more diffuse in ESCs under the microscope (Mayer et al. 2005; Meshorer et al. 2006). This is possibly the consequence of the unusual behavior of classic pericentromeric proteins in ESCs, which are either absent from chromocenters or bind more loosely (Meshorer et al. 2006; Brown et al. 2013). During ESC fusion-mediated reprogramming of somatic nuclei, these atypical features are quickly adopted by the somatic nuclei (Brown et al. 2013), and reprogramming by nuclear transfer is also accompanied by dispersion of chromocenters in the donor nucleus (Martin et al. 2006). Although the disorganized heterochromatic compartment could potentially be explained by the unusually fast proliferation rate of ESCs, similarly disorganized heterochromatin was found in more slowly dividing human ESCs (E de Wit, BA Bouwman, and W de Laat, unpubl.). Thus, the unique chromocenter configuration and PAD organization in ESCs could potentially be fundamental to their pluripotency.

### Cause and consequence of pericentromeric recruitment

The strong global correlation between pericentromeric association and gene repression is consistent with DNA FISH-based notions that PCH in chromocenters comprises a universal repressive compartment in the nucleus. Although convincing, such correlations do not resolve the causal relationship between gene repression and pericentromeric proximity. Our finding that many differentiation-induced PADs are already repressed prior to association with chromocenters suggests that pericentromeric association is not the cause of their repression. They are either silenced due to their proximity to the nuclear periphery, or, as we consider more likely, they are silenced autonomously irrespective of their exact nuclear location. As has been proposed previously (Misteli 2007; Gibcus and Dekker 2013; Krijger and de Laat 2013), the self-organizing principles of chromatin promote an energetically favorable 3D chromatin structure, so that after each mitotic exit, these silent loci will preferentially aggregate with other repressed chromosomal regions to form a silenced compartment. In genome-wide 4C-based interaction maps, this spatial segregation between euchromatin and heterochromatin was already noted (Simonis et al. 2006), and in Hi-C data, this feature is apparent as the A and B compartments (Lieberman-Aiden et al. 2009; Zhang et al. 2012). In most cell types, the silenced compartment is positioned at the periphery, but in rod photoreceptor cells it is placed in the nuclear interior (Solovei et al. 2009). Given that chromocenters aggregate in the nuclear center or at the nuclear periphery depending on the location of inactive chromatin, we propose that for many silenced genomic regions the association with pericentromeric satellite repeats might actually be the consequence of their autonomous clustering at cell-specific nuclear locations. Provided that the G1 phase of the cell cycle allows sufficient time for genomic loci to adopt their most favorable positions, chromocenters will preferentially co-occupy the same inactive compartments, where they come in contact with resident genomic regions. However, if inactive chromatin is spatially disorganized as in pluripotent stem cells, this also leads to a more dispersed nuclear distribution of chromocenters.

### Potential functional contribution of pericentromere association

What, then, is the function of pericentromeric association? By experimentally tethering an active locus to chromocenters, we established a causal role of pericentromeric proximity in the transcriptional repression of associated loci. The resulting repression is accompanied by nucleation of a local heterochromatin domain of the same type as that of pericentromeric heterochromatin itself, not by the H3K9me2 mark that is associated with repression at the nuclear periphery. Repression and relocation to the inactive nuclear compartment may therefore be mediated by both H3K9me2- and H3K9me3-centered pathways, which are not necessarily mutually exclusive.

As mentioned above, LADs do not invariably localize at the nuclear periphery. Kind et al. (2013) recently showed that only ∼30% of LADs return to the nuclear periphery after mitosis. Based on the extensive overlap between LADs and PADs, we hypothesize that silenced loci in the nuclear interior aggregate and coassociate with chromocenters which may help maintain their silenced chromatin state. Consistent with this, dissociation from the lamina in Lamin A-mutant human cell lines did not necessarily lead to gene activation (Kubben et al. 2012). Moreover, mutations in the lamin B receptor and lamin A genes led to a loss of peripheral heterochromatin in post-mitotic mouse cells (Solovei et al. 2013). In these cells, where heterochromatin centers around pericentromeric satellite repeats in the nuclear interior, no large-scale transcriptional changes were detected (Solovei et al. 2013). Thus, gene repression and the spatial segregation of active and inactive chromatin can be maintained in a radial position-independent manner. We propose that chromocenters positioned in the inactive compartment can help maintain silencing independent of radial nuclear location.

Finally, it should be mentioned that similar to induced recruitment to the nuclear lamina (Finlan et al. 2008; Kumaran and Spector 2008; Reddy et al. 2008), recruitment to pericentromeric satellite repeats may not necessarily lead to gene repression as seen with our reporter construct. In *Drosophila*, many protein-coding genes are embedded in pericentromeric heterochromatin (Hoskins et al. 2002), some of which rely on heterochromatic proteins for expression (Lu et al. 2000; Yasuhara and Wakimoto 2006). We also found hundreds of active genes residing in PADs (data not shown). Although these may be expressed only from alleles that are not associated with satellites, we suspect that subsets of genes may be impervious to PCH-induced repression or may have evolved to benefit from proximity to chromocenters.

## Methods

### Primary thymus tissue

Thymus tissue was isolated from adult male mice (C57BL/6), with all experimental procedures approved by the animal welfare committee (DEC) of the Royal Dutch academy of sciences (KNAW). Immediately after collection, thymus tissue was disaggregated through a nylon cell strainer and crosslinked for 10 min (room temperature unless stated otherwise) with 1% formaldehyde at 10 million cells per 10 mL. After addition of glycine to 125 mM, cells were pelleted for 8 min at 600*g* (4°C), washed once in sort buffer (PBS enriched with 25 mM Hepes, 1 mM EDTA, and 1% heat-inactivated fetal bovine serum), and incubated on ice for 10 min in lysis buffer (50 mM Tris pH 7.5, 150 mM NaCl, 5 mM EDTA, 0.5% NP40, 1% Triton X-100, 1× protease inhibitor cocktail). Nuclei were then pelleted for 8 min at 600*g* (4°C), snap-frozen in liquid nitrogen, and stored at −80°C until use.

### Cell culture

Mouse embryonic stem cells (IB10) and neural precursor cells derived from these ESCs were a gift from Bas van Steensel and maintained as previously described (Peric-Hupkes et al. 2010). Astrocytes were derived from these NPCs in our own laboratory according to established protocols (Peric-Hupkes et al. 2010). Approximately 10 million cells were harvested by trypsin treatment and crosslinked as described above.

### Generation of *lacO* transgenic cells

The pLau43 plasmid (Lau et al. 2003) carrying a loxP-flanked neomycin resistance cassette was a gift from Roland Kanaar. The *mCherry* gene with an upstream simian *CMV IE94* promoter was PCR-amplified from a pCS2-*mCherry* plasmid (a gift from Stefan Schultemerker) with primers carrying a NheI overhang and cloned into the XbaI site between the *lacO* repeats and the neomycin resistance cassette. This plasmid was linearized and randomly integrated into the genome of polymorphic *Mus musculus*/*Mus castaneus* embryonic stem cells carrying an ms2-tagged *Xist* ([Jonkers et al. 2008], a gift from Joost Gribnau) using standard procedures. Integration sites of 56 colonies were identified by nested PCR using transgene-specific primers and a partially random primer as described (Ruf et al. 2011), followed by Sanger sequencing. *LacO* tethering experiments were performed on clone 36 (integration site Chr 11: 115,708,347). This integration site was independently validated by 4C-seq analysis from the *lacO* cassette (data not shown).

### LacR-fusion constructs

To express EGFP-LacR fusion proteins under control of the *EEF1A1* promoter, we replaced the *DsRed* gene of the phage2-EEF1A1-DsRed-IRES vector (gift from Niels Geijsen) by the coding regions of *EGFP-lacR* (a gift from Pernette Verschure) using the NotI and BamHI sites. The chromodomain was PCR-amplified from full-length *Cbx1* and put behind *lacR*. The threonine-to-alanine mutation at residue 34 of the chromodomain was done using the QuickChange site-directed mutagenesis II kit (Stratagene). Transgenic cells were transfected using the Amaxa nucleofection kit (Lonza) as detailed in experimental procedures. GFP-positive cells were FACS-sorted 72 h after nucleofection on a FACSAria (BD Biosciences) while simultaneously measuring mCherry expression. For chromatin immunoprecipitation, cells were transduced with lentiviruses of the same constructs and expanded for 10 d under puromycin selection (see Supplemental Methods for details).

### Nucleofections and transductions

For transient transfections, *lacO*-transgenic ESCs were grown on plates coated with 0.15% gelatin in the presence of G418. After refreshing media 4 h prior, an Amaxa Nucleofector (Lonza) was used to transfect 10-cm plates of cells at 80% confluency. Cells were trypsinized and made into single cell suspensions before being spun down at 200*g* for 4 min. The media was aspirated and the cells taken up in 5 mL PBS and spun again at 200*g* for 4 min. Cells were aspirated again and taken up in 90 µL nucleofection buffer (90 mM sodium phosphate buffer pH 7.2, 5 mM KCL, 10 mM MgCl_2_, 20 mM Hepes-KOH pH 7.2, 24 mM Na succinate, adjusted to pH 7.2). Cells were then mixed with 10 µL nucleofection buffer containing 20 µg of plasmid DNA and nucleofected in electroporation cuvettes using program A-23 (mouse ES cells). Immediately after nucleofection, 0.5 mL warm media was added, and the cells were transferred to a 15-cm plate with 20 mL of media (from this point, G418 was left out of the media). Media was refreshed ∼20, 44, and 68 h after transfection, with Puromycin (P8833, Sigma) added at 1 µg/µL from 44 h onwards. Cells were analyzed ∼72 h post-nucleofection.

To produce enough cells for ChIP, *lacO*-transgenic ESCs were transduced with *EGFP-lacR* fusions using lentivirus based on the pHAGE2-IRES-puro backbone with an *EEF1A1* promoter (Wilson et al. 2008). GFP-positive cells were selected with puromycin for 8–10 d, after which cells were collected and tested for purity by flow cytometry (all >80% GFP-positive) (data not shown). Expression of the mCherry reporter upon transduction gave similar results to transient transfection (data not shown).

### Fluorescence-activated cell sorting

Sorting was done 72 h after nucleofection; cells were made into single-cell suspensions before being spun down for 4 min at 200*g*, aspirated, and then taken up in sort buffer. GFP-positive cells were sorted on a FACSAria (BD Biosciences) while simultaneously measuring mCherry expression. Sorted cells were spotted on poly-L-lysine coated glass slides and crosslinked with 3% paraformaldehyde in PBS for 10 min. After one PBS wash, cells were permeabilized in ice-cold 0.5% Triton X-100 in PBS for 6 min, followed by two 5-min washes with 70% ethanol in which they were stored at −20°C until use in fluorescent in situ hybridization (FISH).

### GFP distribution analysis

To analyze the distribution of EGFP-LacR fusion proteins, FACS-sorted GFP^+^ cells were grown overnight on gelatin-coated coverslips. The following day, cells were crosslinked for 10 min in 4% paraformaldehyde, washed once with 0.125 M glycine in PBS, and permeabilized 5 min with 0.2% Triton X-100 in PBS. After one wash in PBS containing 0.1% Tween 20, coverslips were mounted in VectaShield containing DAPI (Vector Labs) and sealed with nail varnish. Images were taken on a Leica SPE confocal microscope and analyzed using ImageJ software.

### DNA fluorescent in situ hybridization

Primary thymus and FACS-sorted cells were crosslinked on poly-L-lysine coated glass slides as described above. For probe labeling, the *lacO* transgene and a bacterial artificial chromosome (BAC) across the *Llgl2* locus (RP23-143F14) were fragmented with Sau3A, while major satellite repeats were PCR-amplified from diluted genomic DNA to obtain fragments corresponding to 1–3 repeats. Probes were labeled as previously described (Splinter et al. 2011) with the BAC, *lacO* transgene, and satellites labeled in green, Cy3 and Cy5, respectively (Cat. #42845, 42501, and 42502, Enzo Life Sciences). For hybridizations, 5 µL of satellite and *lacO* probe and 10 µL BAC probe were combined with 5 µL mouse Cot1 (18440-016, Invitrogen), speedvacced until dry, and dissolved in 12.5 µL 50+ Hybmix (50% deionized formamide [F9037, Sigma], 2× SSC, 2.5× Denhardt's [750018, Invitrogen], 1 mM EDTA, 50% dextran sulphate [17-0340-01, GE Healthcare]). Probes were denatured for 5 min at 95°C, chilled for 5 min, and pre-annealed for 30 min at 37°C. During probe preparation, FISH slides were sequentially dehydrated in 70%, 90%, and 100% ethanol (2 min each). The slides were air-dried briefly and denatured on a heat block for 3 min at 85°C in 100 µL of 70+ Hybmix (70% deionized formamide, 2× SSC, 10 mM phosphate buffer pH 7.0). Slides were immediately washed twice in prechilled 2× SSC on ice, dehydrated in ethanol series, and air-dried for 5 min. Ten microliters of probe mix was applied to each slide, and hybridization took place for 72 h in a dark humidified box at 37°C. Slides were then washed three times in 50% formamide/2× SSC and two times in 2× SSC (all 5 min at 45°C), stained for 2 min with DAPI (2 ng/mL in 2× SSC), and washed twice more in 2× SSC for 5 min each. Finally, slides were mounted in 10 µL Prolong gold (P36930, Invitrogen) and a coverslip sealed in place using nail varnish. DNA FISH for validation of sat4C using eight BACs (details below) was performed in the same way but with all BACs labeled in green and only in combination with Cy5-labeled major satellites.

### Image acquisition

Three-dimensional images for distance measurements were taken on a Leica DM6000 fluorescent microscope (Leica Microsystems) with a DFC360FX-325642208 camera and a HCX PL APO CS 100.0×1.40 oil objective (voxel size 128:128:148 nm [x:y:z]). Distances were measured manually from the center of each BAC signal to the edge of the closest chromocenter (marked by satellite probe) using ImageJ software as previously described (Splinter et al. 2011). Association was called when distances between the center of the BAC signal and the edge of the closest chromocenter were <0.3 μm, the maximum distance at which visual associations (touching signals) have been observed in our measurements. For comparative measurements in *lacO* transgenic cells, Z-stacks were renamed in a randomized fashion to allow unbiased measurements, and only cells were included that showed two BAC spots and one overlapping *lacO* spot.

Imaging of EGFP-LacR fusions was performed on a Leica TCS SPE spectral confocal microscope (Leica Microsystems) using an ACS APO 63.0×1.30 oil objective with 3.0× zoom (voxel size 56.9:56.9:209.8 nm [x:y:z]).

### Chromatin immunoprecipitation

Transduced cell populations were harvested and made into single cell suspensions, before equal cell numbers were used for ChIP with antibodies against H3K9me2 (ab1220, Abcam) and H3K9me3 (ab8898, Abcam). ChIP was essentially performed as previously described (Schmidt et al. 2009) with 2.5 million cells per IP. Quantitative PCR (see Supplemental Material for primer sequences) was performed and data normalized to the *Actb* promoter. To control for differences in IP efficiencies across three independent ChIP experiments, enrichment values were calculated relative to EGFP-LacR of each replicate experiment.

### Sat4C procedure

To overcome sequencing of thousands of satellite-to-satellite ligation events, we modified the standard 4C protocol (Splinter et al. 2012) in several ways. The principle difference is that we fragmented the genome with a combination of ApoI (fragments the whole genome, including most major satellite repeats) and MfeI (cuts throughout the genome, but not in major satellites). Subsequent ligation events between 5′ AATT overhangs of ApoI-digested major satellite repeats and MfeI-digested genomic fragments are enriched through three means: (1) Self-ligations between ApoI-digested satellite fragments are redigested with ApoI before inverse PCR enrichment to prevent their amplification; (2) primers with a 3′ end of AATTG favor amplification of ApoI-MfeI ligation events at higher annealing temperatures (Supplemental Material); and (3) an additional AcuI digest to fragment satellite repeats, further preventing amplification of (ApoI-uncut) satellite multimers while leaving ligations of interest intact.

In general, sat4C follows the regular 4C protocol as detailed by Splinter et al. (2012) with several modifications. Cells were crosslinked with 1% formaldehyde, rather than 2%, as MfeI digestion is impaired by higher formaldehyde concentrations. Crosslinked nuclei (described above) were washed once in 1× NEBuffer 4 and resuspended in 429 µL with 60 µL 10× NEBuffer 4. Nuclei were left shaking at 37°C for 2 h with the addition of 15 µL 10% SDS after 2 min and 50 µL 20% Triton X-100 after 1 h. Nuclei were digested with 400 units ApoI (NEB) for 2 h at 50°C followed by an overnight incubation at 37°C while shaking. After confirming ApoI digestion (as detailed in Splinter et al. 2012), 200 units MfeI were added in three rounds (morning, evening, morning) with addition of fresh NEBuffer 4. Ligation was performed as overnight at 16°C in 7 mL with 20 units T4 DNA ligase. After overnight reverse crosslinking at 65°C with 300 µg Proteinase K, samples were incubated for 45 min with 300 µg RNase A (37°C), and DNA was purified with phenol-chloroform and ethanol precipitation (Splinter et al. 2012). Samples were subsequently digested overnight with 50 units NlaIII (NEB) in a 500-µL reaction, NlaIII was inactivated for 20 min at 65°C, and ligation was again performed as previously described (Splinter et al. 2012). After DNA purification, 4C circles were redigested with 50 units ApoI (4 h at 50°C) and 100 units AcuI (overnight at 37°C) before a final round of purification over QIAquick columns (Qiagen). Sixteen 25-µL PCR reactions were performed, with each containing 1× ELT buffer (Roche), 0.4 µL ELT polymerase (Roche), 200 µM dNTPS, 150 ng forward primer, 100 ng reverse primer, and 25 ng 4C template. The PCR program consisted of 32 cycles of 20 sec at 95°C, 1 min at 62°C, and 3 min at 68°C. PCR samples were pooled back and purified using Agencourt AMPure XP beads (Beckman Coulter) in a 1:1 ratio. DNA was eluted in 100 µL 10 mM Tris pH 8.0 and was analyzed by next generation sequencing (Illumina).

### Sat4C mapping and normalization

Sat4C data were mapped against a reduced genome (*Mus musculus*, *mm9* excluding the Y chromosome) consisting of sequences flanking 4C restriction sites (referred to as 4C fragment ends) as previously described (van de Werken et al. 2012). To reduce the influence of potential PCR amplification bias, the 0.025% highest observed reads in each experiment were set to the 99.975% quantile. Mapped reads were normalized for sequencing depth by multiplying with a constant, such that the total number of reads is equal in all experiments considered. Normalized sat4C coverage profiles were generated for each individual chromosome by computing the mean number of mapped reads in a running window of 101 4C fragment ends (median size 600 kb). Very similar profiles are obtained with smaller (>21) fragment-end windows (data not shown). To get more intuitive profiles for visualization, we subtracted the chromosome-wide average. To compare across cell types, matrices of genome-wide mean-normalized sat4C profiles were quantile-normalized.

### Domain identification

For each individual replicate experiment, the read coverage per 4C fragment end was binned into the following categories: [0 reads],[1 read],[2–4 reads],[5–7 reads],[>7 reads]. A two-state hidden semi-Markov model (HSMM) was fitted to estimate the probability of observing reads from these categories conditional on the hidden state, i.e., being in a “PAD” or a “non-PAD.” Conditional distributions of the observed reads were assumed to be multinomial, and the so-called sojourn time density was assumed to be gamma. Models were fit in R (R Core Team 2014) using an iterative expectation-maximization (EM) type of approach implemented in the mhsmm CRAN package (O'Connell and Højsgaard 2011). PAD calls at individual 4C fragment ends per tissue or cell type were based on a majority vote across all replicates. In case of ties, another virtual replicate was created by pooling reads from all corresponding replicate experiments. Genomic PAD and non-PAD domains were obtained by taking unions of regions covered by consecutive 4C fragment ends with identical HSMM calls. PAD borders are defined as the centers between consecutive 4C fragment ends with different HSMM calls.

### Statistical analysis

All statistical analyses were performed in R/Bioconductor (Gentleman et al. 2004). Manipulation with and computation of statistics on genomic intervals and domains was done using the GenomicRanges package (Lawrence et al. 2013).

### Pericentromeric association segregates repressed chromatin from active chromatin

The mouse genome was partitioned into consecutive, nonoverlapping 20 kb bins. We computed overlap between the bins and a set of 22,492 nonredundant mm9 RefSeq transcripts and calculated gene density for each 20-kb window as the percentage of bp sequence overlap. The average RNA-seq FPKM gene expression for these transcripts in mouse primary thymus was computed at each window by averaging over all overlapping RefSeq genes. Locations of published ChIP-seq BroadPeaks and DNase I hypersensitive sites (hotspots) (The ENCODE Project Consortium 2011) were overlapped, and the average score of overlapping events was computed. Next, we overlapped H3K9me2 enrichment values from published ChIP-chip data (Lienert et al. 2011) in terminally differentiated neurons and averaged all overlapping probes per bin. We obtained genomic coordinates of domains of H4K20me3 and H3K9me3 enrichment in primary mouse liver (Magklara et al. 2011), based on ChIP-chip data. We calculated the percentage of genomic sequence overlap between these domains with each 20-kb window.

Finally, we overlapped microarray data from replication timing experiments (Weddington et al. 2008) and computed the mean “replication timing” for each bin from overlapping probes. We calculated the distance between the centers of all 20-kb windows to the nearest PAD borders and binned distances in intervals of 125 kb. Then, for all data described above, we averaged the computed quantities at each 20-kb window over all such windows within a given distance-to-PAD-border interval, resulting in a matrix where rows correspond to the different types of data and columns represent binned distance to PAD borders. We visualized this matrix as a “heatmap” (Fig. 2C) with a white-to-green color gradient using the pheatmap package in R.

We downloaded the Hi-C directionality index data from mouse ES cells published by Dixon et al. (2012). This contains genomic intervals of 40 kb at which an index that quantifies asymmetric bias in Hi-C chromatin interactions either up- or downstream is computed. The absolute value of the directionality index is a measure of the tendency of a region to prefer interactions in either direction. We computed the distance of the 40-kb intervals to the nearest PAD border in thymus (as defined above) and binned these distances at intervals of 80 kb. Then we computed the 5% trimmed mean of the absolute value of the directionality index at this binned set of distances and plotted the average magnitude of directionality index against distance to PAD border (Fig. 2E). We repeated the same procedure with borders of TADs. To obtain randomized borders, we shifted PAD borders over a random distance, i.e., a number drawn uniformly at random from the interval [0;6.4 Mb]. We shifted the borders in a circular fashion, such that a border that was 1 Mb away from the 3′ end of the chromosome that was shifted more than 2.5 Mb will end up at 1.5 Mb from the 5′ start of the same chromosome. We used 1000 sets of randomly shifted borders and averaged the results.

Segregation of inactive chromatin around chromocenters is established during lineage commitment. For the identified sets of PAD and non-PAD domains in mouse ESCs, NPCs, and ACs, we calculate the percentage of overlapping clusters of DNase I hypersensitive sites in mouse ESCs. For PADs and non-PADs in thymus, we use DNase I hypersensitivity data from the corresponding tissue. We define constitutive PADs as the consecutive intersection of PAD domains in ESCs, NPCs, ACs, and thymus. ESC facultative PADs and non-PADs in Figure 3F are obtained by intersecting with thymus non-PADs or PADs, respectively.

### Chromocenters progressively overlap with inactive chromatin at the nuclear periphery

For mouse ESCs, NPCs, and ACs, we calculate the genomic overlap between the PADs we have defined in these tissues with published LADs defined in the corresponding cell types. The PADs in thymus are overlapped (genomic overlap in bp) with AC LADs. To quantify the significance of the observed overlap in each cell type, we define randomized PADs that we obtain by 1000 random circular permutations of the HSMM calls at 4C fragment ends. This procedure works as follows. We concatenate the HSMM calls at all 4C fragment ends on all chromosomes into a vector of size *n*, with indices (1,2,…*n*). A “circular permutation” of this vector is obtained by shifting all indices of this vector by a random number *k* in [1,…,*n*], such that the permuted vector consists of elements (*n* − *k* + 1, *n* − *k* + 2,….,*n* − 1,*n*,1,2,…,*n* − *k*) of the original vector. Within each iteration, we draw a new value of *k* uniformly at random and define “randomized PADs” based on a circularly permuted vector of HSMM calls and calculate the overlap between these randomized PADs and LADs from the corresponding cell type. The expected values in Figure 3G are the means of the corresponding distributions of randomized overlaps, and the bars are the observed 5% and 95% quantiles of this distribution.

## Data access

Sat4C data for thymus, ESC, NPC, and AC from this study have been submitted to the NCBI Gene Expression Omnibus (GEO; http://www.ncbi.nlm.nih.gov/geo/) under accession number GSE65618.

## References

[WIJCHERSGR186643C1] Alcobia I, Dilao R, Parreira L. 2000 Spatial associations of centromeres in the nuclei of hematopoietic cells: evidence for cell-type-specific organizational patterns. Blood 95: 1608–1615. 10688815

[WIJCHERSGR186643C2] Ayoub N, Jeyasekharan AD, Bernal JA, Venkitaraman AR. 2008 HP1-β mobilization promotes chromatin changes that initiate the DNA damage response. Nature 453: 682–686. 18438399 10.1038/nature06875

[WIJCHERSGR186643C3] Ayyanathan K, Lechner MS, Bell P, Maul GG, Schultz DC, Yamada Y, Tanaka K, Torigoe K, Rauscher FJ III. 2003 Regulated recruitment of HP1 to a euchromatic gene induces mitotically heritable, epigenetic gene silencing: a mammalian cell culture model of gene variegation. Genes Dev 17: 1855–1869. 12869583 10.1101/gad.1102803 PMC196232

[WIJCHERSGR186643C4] Baccarini P. 1908 Sulle cinesi vegetative del *Cynomorium coccineum L*. Nuovo Giorn Bot Ital 15: 189–203.

[WIJCHERSGR186643C5] Bannister AJ, Zegerman P, Partridge JF, Miska EA, Thomas JO, Allshire RC, Kouzarides T. 2001 Selective recognition of methylated lysine 9 on histone H3 by the HP1 chromo domain. Nature 410: 120–124. 11242054 10.1038/35065138

[WIJCHERSGR186643C6] Bartholdi MF. 1991 Nuclear distribution of centromeres during the cell cycle of human diploid fibroblasts. J Cell Sci 99: 255–263. 1885670 10.1242/jcs.99.2.255

[WIJCHERSGR186643C7] Beil M, Durschmied D, Paschke S, Schreiner B, Nolte U, Bruel A, Irinopoulou T. 2002 Spatial distribution patterns of interphase centromeres during retinoic acid-induced differentiation of promyelocytic leukemia cells. Cytometry 47: 217–225. 11933011 10.1002/cyto.10077

[WIJCHERSGR186643C8] Bickmore WA, van Steensel B. 2013 Genome architecture: domain organization of interphase chromosomes. Cell 152: 1270–1284. 23498936 10.1016/j.cell.2013.02.001

[WIJCHERSGR186643C9] Brown KE, Guest SS, Smale ST, Hahm K, Merkenschlager M, Fisher AG. 1997 Association of transcriptionally silent genes with Ikaros complexes at centromeric heterochromatin. Cell 91: 845–854. 9413993 10.1016/s0092-8674(00)80472-9

[WIJCHERSGR186643C10] Brown KE, Baxter J, Graf D, Merkenschlager M, Fisher AG. 1999 Dynamic repositioning of genes in the nucleus of lymphocytes preparing for cell division. Mol Cell 3: 207–217. 10078203 10.1016/s1097-2765(00)80311-1

[WIJCHERSGR186643C11] Brown KE, Amoils S, Horn JM, Buckle VJ, Higgs DR, Merkenschlager M, Fisher AG. 2001 Expression of α- and β-globin genes occurs within different nuclear domains in haemopoietic cells. Nat Cell Biol 3: 602–606. 11389446 10.1038/35078577

[WIJCHERSGR186643C12] Brown KE, Bagci H, Soza-Ried J, Fisher AG. 2013 Atypical heterochromatin organization and replication are rapidly acquired by somatic cells following fusion-mediated reprogramming by mouse ESCs. Cell Cycle 12: 3253–3261. 24036550 10.4161/cc.26223 PMC3885636

[WIJCHERSGR186643C13] de Koning AP, Gu W, Castoe TA, Batzer MA, Pollock DD. 2011 Repetitive elements may comprise over two-thirds of the human genome. PLoS Genet 7: e1002384. 22144907 10.1371/journal.pgen.1002384 PMC3228813

[WIJCHERSGR186643C14] de Laat W, Duboule D. 2013 Topology of mammalian developmental enhancers and their regulatory landscapes. Nature 502: 499–506. 24153303 10.1038/nature12753

[WIJCHERSGR186643C16] de Wit E, Bouwman BA, Zhu Y, Klous P, Splinter E, Verstegen MJ, Krijger PH, Festuccia N, Nora EP, Welling M, 2013 The pluripotent genome in three dimensions is shaped around pluripotency factors. Nature 501: 227–231. 23883933 10.1038/nature12420

[WIJCHERSGR186643C15] Deniaud E, Bickmore WA. 2009 Transcription and the nuclear periphery: edge of darkness? Curr Opin Genet Dev 19: 187–191. 19231154 10.1016/j.gde.2009.01.005

[WIJCHERSGR186643C17] Dixon JR, Selvaraj S, Yue F, Kim A, Li Y, Shen Y, Hu M, Liu JS, Ren B. 2012 Topological domains in mammalian genomes identified by analysis of chromatin interactions. Nature 485: 376–380. 22495300 10.1038/nature11082 PMC3356448

[WIJCHERSGR186643C18] Eberhart A, Feodorova Y, Song C, Wanner G, Kiseleva E, Furukawa T, Kimura H, Schotta G, Leonhardt H, Joffe B, 2013 Epigenetics of eu- and heterochromatin in inverted and conventional nuclei from mouse retina. Chromosome Res 21: 535–554. 23996328 10.1007/s10577-013-9375-7

[WIJCHERSGR186643C19] The ENCODE Project Consortium. 2011 A user's guide to the encyclopedia of DNA elements (ENCODE). PLoS Biol 9: e1001046. 21526222 10.1371/journal.pbio.1001046 PMC3079585

[WIJCHERSGR186643C20] Finlan LE, Sproul D, Thomson I, Boyle S, Kerr E, Perry P, Ylstra B, Chubb JR, Bickmore WA. 2008 Recruitment to the nuclear periphery can alter expression of genes in human cells. PLoS Genet 4: e1000039. 18369458 10.1371/journal.pgen.1000039 PMC2265557

[WIJCHERSGR186643C21] Francastel C, Walters MC, Groudine M, Martin DI. 1999 A functional enhancer suppresses silencing of a transgene and prevents its localization close to centrometric heterochromatin. Cell 99: 259–269. 10555142 10.1016/s0092-8674(00)81657-8

[WIJCHERSGR186643C22] Fransz P, De Jong JH, Lysak M, Castiglione MR, Schubert I. 2002 Interphase chromosomes in *Arabidopsis* are organized as well defined chromocenters from which euchromatin loops emanate. Proc Natl Acad Sci 99: 14584–14589. 12384572 10.1073/pnas.212325299 PMC137926

[WIJCHERSGR186643C23] Funabiki H, Hagan I, Uzawa S, Yanagida M. 1993 Cell cycle-dependent specific positioning and clustering of centromeres and telomeres in fission yeast. J Cell Biol 121: 961–976. 8388878 10.1083/jcb.121.5.961 PMC2119680

[WIJCHERSGR186643C24] Gentleman RC, Carey VJ, Bates DM, Bolstad B, Dettling M, Dudoit S, Ellis B, Gautier L, Ge Y, Gentry J, 2004 Bioconductor: open software development for computational biology and bioinformatics. Genome Biol 5: R80. 15461798 10.1186/gb-2004-5-10-r80 PMC545600

[WIJCHERSGR186643C25] Gibcus JH, Dekker J. 2013 The hierarchy of the 3D genome. Mol Cell 49: 773–782. 23473598 10.1016/j.molcel.2013.02.011 PMC3741673

[WIJCHERSGR186643C26] Gregory TR. 2005 Synergy between sequence and size in large-scale genomics. Nat Rev Genet 6: 699–708. 16151375 10.1038/nrg1674

[WIJCHERSGR186643C27] Guelen L, Pagie L, Brasset E, Meuleman W, Faza MB, Talhout W, Eussen BH, de Klein A, Wessels L, de Laat W, 2008 Domain organization of human chromosomes revealed by mapping of nuclear lamina interactions. Nature 453: 948–951. 18463634 10.1038/nature06947

[WIJCHERSGR186643C28] Hathaway NA, Bell O, Hodges C, Miller EL, Neel DS, Crabtree GR. 2012 Dynamics and memory of heterochromatin in living cells. Cell 149: 1447–1460. 22704655 10.1016/j.cell.2012.03.052 PMC3422694

[WIJCHERSGR186643C29] Hewitt SL, High FA, Reiner SL, Fisher AG, Merkenschlager M. 2004 Nuclear repositioning marks the selective exclusion of lineage-inappropriate transcription factor loci during T helper cell differentiation. Eur J Immunol 34: 3604–3613. 15484194 10.1002/eji.200425469

[WIJCHERSGR186643C30] Hiragami K, Festenstein R. 2005 Heterochromatin protein 1: a pervasive controlling influence. Cell Mol Life Sci 62: 2711–2726. 16261261 10.1007/s00018-005-5287-9 PMC11139183

[WIJCHERSGR186643C31] Hon GC, Rajagopal N, Shen Y, McCleary DF, Yue F, Dang MD, Ren B. 2013 Epigenetic memory at embryonic enhancers identified in DNA methylation maps from adult mouse tissues. Nat Genet 45: 1198–1206. 23995138 10.1038/ng.2746 PMC4095776

[WIJCHERSGR186643C32] Hoskins RA, Smith CD, Carlson JW, Carvalho AB, Halpern A, Kaminker JS, Kennedy C, Mungall CJ, Sullivan BA, Sutton GG, 2002 Heterochromatic sequences in a *Drosophila* whole-genome shotgun assembly. Genome Biol 3: RESEARCH0085. 12537574 10.1186/gb-2002-3-12-research0085 PMC151187

[WIJCHERSGR186643C33] Jonkers I, Monkhorst K, Rentmeester E, Grootegoed JA, Grosveld F, Gribnau J. 2008 Xist RNA is confined to the nuclear territory of the silenced X chromosome throughout the cell cycle. Mol Cell Biol 28: 5583–5594. 18625719 10.1128/MCB.02269-07 PMC2546918

[WIJCHERSGR186643C34] Jost KL, Haase S, Smeets D, Schrode N, Schmiedel JM, Bertulat B, Herzel H, Cremer M, Cardoso MC. 2011 3D-Image analysis platform monitoring relocation of pluripotency genes during reprogramming. Nucleic Acids Res 39: e113. 21700670 10.1093/nar/gkr486 PMC3177216

[WIJCHERSGR186643C35] Kind J, Pagie L, Ortabozkoyun H, Boyle S, de Vries SS, Janssen H, Amendola M, Nolen LD, Bickmore WA, van Steensel B. 2013 Single-cell dynamics of genome-nuclear lamina interactions. Cell 153: 178–192. 23523135 10.1016/j.cell.2013.02.028

[WIJCHERSGR186643C36] Krijger PH, de Laat W. 2013 Identical cells with different 3D genomes; cause and consequences? Curr Opin Genet Dev 23: 191–196. 23415810 10.1016/j.gde.2012.12.010

[WIJCHERSGR186643C37] Kubben N, Adriaens M, Meuleman W, Voncken JW, van Steensel B, Misteli T. 2012 Mapping of lamin A- and progerin-interacting genome regions. Chromosoma 121: 447–464. 22610065 10.1007/s00412-012-0376-7 PMC3443488

[WIJCHERSGR186643C38] Kumaran RI, Spector DL. 2008 A genetic locus targeted to the nuclear periphery in living cells maintains its transcriptional competence. J Cell Biol 180: 51–65. 18195101 10.1083/jcb.200706060 PMC2213611

[WIJCHERSGR186643C39] Lachner M, O'Carroll D, Rea S, Mechtler K, Jenuwein T. 2001 Methylation of histone H3 lysine 9 creates a binding site for HP1 proteins. Nature 410: 116–120. 11242053 10.1038/35065132

[WIJCHERSGR186643C40] Lau IF, Filipe SR, Soballe B, Okstad OA, Barre FX, Sherratt DJ. 2003 Spatial and temporal organization of replicating *Escherichia coli* chromosomes. Mol Microbiol 49: 731–743. 12864855 10.1046/j.1365-2958.2003.03640.x

[WIJCHERSGR186643C41] Lawrence M, Huber W, Pages H, Aboyoun P, Carlson M, Gentleman R, Morgan MT, Carey VJ. 2013 Software for computing and annotating genomic ranges. PLoS Comput Biol 9: e1003118. 23950696 10.1371/journal.pcbi.1003118 PMC3738458

[WIJCHERSGR186643C42] Lieberman-Aiden E, van Berkum NL, Williams L, Imakaev M, Ragoczy T, Telling A, Amit I, Lajoie BR, Sabo PJ, Dorschner MO, 2009 Comprehensive mapping of long-range interactions reveals folding principles of the human genome. Science 326: 289–293. 19815776 10.1126/science.1181369 PMC2858594

[WIJCHERSGR186643C43] Lienert F, Mohn F, Tiwari VK, Baubec T, Roloff TC, Gaidatzis D, Stadler MB, Schubeler D. 2011 Genomic prevalence of heterochromatic H3K9me2 and transcription do not discriminate pluripotent from terminally differentiated cells. PLoS Genet 7: e1002090. 21655081 10.1371/journal.pgen.1002090 PMC3107198

[WIJCHERSGR186643C44] Lu BY, Emtage PC, Duyf BJ, Hilliker AJ, Eissenberg JC. 2000 Heterochromatin protein 1 is required for the normal expression of two heterochromatin genes in *Drosophila*. Genetics 155: 699–708. 10835392 10.1093/genetics/155.2.699 PMC1461102

[WIJCHERSGR186643C45] Lundgren M, Chow CM, Sabbattini P, Georgiou A, Minaee S, Dillon N. 2000 Transcription factor dosage affects changes in higher order chromatin structure associated with activation of a heterochromatic gene. Cell 103: 733–743. 11114330 10.1016/s0092-8674(00)00177-x

[WIJCHERSGR186643C46] Magklara A, Yen A, Colquitt BM, Clowney EJ, Allen W, Markenscoff-Papadimitriou E, Evans ZA, Kheradpour P, Mountoufaris G, Carey C, 2011 An epigenetic signature for monoallelic olfactory receptor expression. Cell 145: 555–570. 21529909 10.1016/j.cell.2011.03.040 PMC3094500

[WIJCHERSGR186643C47] Manuelidis L. 1984 Different central nervous system cell types display distinct and nonrandom arrangements of satellite DNA sequences. Proc Natl Acad Sci 81: 3123–3127. 6587343 10.1073/pnas.81.10.3123 PMC345233

[WIJCHERSGR186643C80] Martens JH, O'Sullivan RJ, Braunschweig U, Opravil S, Radolf M, Steinlein P, Jenuwein T. 2005 The profile of repeat-associated histone lysine methylation states in the mouse epigenome. EMBO J 24: 800–812. 15678104 10.1038/sj.emboj.7600545 PMC549616

[WIJCHERSGR186643C48] Martin C, Brochard V, Migne C, Zink D, Debey P, Beaujean N. 2006 Architectural reorganization of the nuclei upon transfer into oocytes accompanies genome reprogramming. Mol Reprod Dev 73: 1102–1111. 16736527 10.1002/mrd.20506

[WIJCHERSGR186643C49] Mayer R, Brero A, von Hase J, Schroeder T, Cremer T, Dietzel S. 2005 Common themes and cell type specific variations of higher order chromatin arrangements in the mouse. BMC Cell Biol 6: 44. 16336643 10.1186/1471-2121-6-44 PMC1325247

[WIJCHERSGR186643C50] Meshorer E, Yellajoshula D, George E, Scambler PJ, Brown DT, Misteli T. 2006 Hyperdynamic plasticity of chromatin proteins in pluripotent embryonic stem cells. Dev Cell 10: 105–116. 16399082 10.1016/j.devcel.2005.10.017 PMC1868458

[WIJCHERSGR186643C51] Misteli T. 2007 Beyond the sequence: cellular organization of genome function. Cell 128: 787–800. 17320514 10.1016/j.cell.2007.01.028

[WIJCHERSGR186643C52] Nakayama J, Rice JC, Strahl BD, Allis CD, Grewal SI. 2001 Role of histone H3 lysine 9 methylation in epigenetic control of heterochromatin assembly. Science 292: 110–113. 11283354 10.1126/science.1060118

[WIJCHERSGR186643C53] Nora EP, Lajoie BR, Schulz EG, Giorgetti L, Okamoto I, Servant N, Piolot T, van Berkum NL, Meisig J, Sedat J, 2012 Spatial partitioning of the regulatory landscape of the X-inactivation centre. Nature 485: 381–385. 22495304 10.1038/nature11049 PMC3555144

[WIJCHERSGR186643C54] O'Connell J, Højsgaard S. 2011 Hidden semi Markov models for multiple observation sequences: the mhsmm package for R. J Stat Softw 39: 1–22.21572908

[WIJCHERSGR186643C55] Peric-Hupkes D, Meuleman W, Pagie L, Bruggeman SW, Solovei I, Brugman W, Graf S, Flicek P, Kerkhoven RM, van Lohuizen M, 2010 Molecular maps of the reorganization of genome-nuclear lamina interactions during differentiation. Mol Cell 38: 603–613. 20513434 10.1016/j.molcel.2010.03.016 PMC5975946

[WIJCHERSGR186643C56] Politz JC, Scalzo D, Groudine M. 2013 Something silent this way forms: the functional organization of the repressive nuclear compartment. Annu Rev Cell Dev Biol 29: 241–270. 23834025 10.1146/annurev-cellbio-101512-122317 PMC3999972

[WIJCHERSGR186643C57] Probst AV, Almouzni G. 2008 Pericentric heterochromatin: dynamic organization during early development in mammals. Differentiation 76: 15–23. 17825083 10.1111/j.1432-0436.2007.00220.x

[WIJCHERSGR186643C58] R Core Team. 2014 R: a language and environment for statistical computing. R Foundation for Statistical Computing, Vienna, Austria http://www.R-project.org/.

[WIJCHERSGR186643C59] Reddy KL, Zullo JM, Bertolino E, Singh H. 2008 Transcriptional repression mediated by repositioning of genes to the nuclear lamina. Nature 452: 243–247. 18272965 10.1038/nature06727

[WIJCHERSGR186643C60] Robinett CC, Straight A, Li G, Willhelm C, Sudlow G, Murray A, Belmont AS. 1996 In vivo localization of DNA sequences and visualization of large-scale chromatin organization using lac operator/repressor recognition. J Cell Biol 135: 1685–1700. 8991083 10.1083/jcb.135.6.1685 PMC2133976

[WIJCHERSGR186643C61] Ruf S, Symmons O, Uslu VV, Dolle D, Hot C, Ettwiller L, Spitz F. 2011 Large-scale analysis of the regulatory architecture of the mouse genome with a transposon-associated sensor. Nat Genet 43: 379–386. 21423180 10.1038/ng.790

[WIJCHERSGR186643C62] Sabbattini P, Lundgren M, Georgiou A, Chow C, Warnes G, Dillon N. 2001 Binding of Ikaros to the λ5 promoter silences transcription through a mechanism that does not require heterochromatin formation. EMBO J 20: 2812–2822. 11387214 10.1093/emboj/20.11.2812 PMC125479

[WIJCHERSGR186643C63] Schmidt D, Wilson MD, Spyrou C, Brown GD, Hadfield J, Odom DT. 2009 ChIP-seq: using high-throughput sequencing to discover protein-DNA interactions. Methods 48: 240–248. 19275939 10.1016/j.ymeth.2009.03.001 PMC4052679

[WIJCHERSGR186643C64] Schubeler D, Francastel C, Cimbora DM, Reik A, Martin DI, Groudine M. 2000 Nuclear localization and histone acetylation: a pathway for chromatin opening and transcriptional activation of the human β-globin locus. Genes Dev 14: 940–950. 10783166 PMC316536

[WIJCHERSGR186643C65] Simonis M, Klous P, Splinter E, Moshkin Y, Willemsen R, de Wit E, van Steensel B, de Laat W. 2006 Nuclear organization of active and inactive chromatin domains uncovered by chromosome conformation capture-on-chip (4C). Nat Genet 38: 1348–1354. 17033623 10.1038/ng1896

[WIJCHERSGR186643C66] Solovei I, Kreysing M, Lanctot C, Kosem S, Peichl L, Cremer T, Guck J, Joffe B. 2009 Nuclear architecture of rod photoreceptor cells adapts to vision in mammalian evolution. Cell 137: 356–368. 19379699 10.1016/j.cell.2009.01.052

[WIJCHERSGR186643C67] Solovei I, Wang AS, Thanisch K, Schmidt CS, Krebs S, Zwerger M, Cohen TV, Devys D, Foisner R, Peichl L, 2013 LBR and lamin A/C sequentially tether peripheral heterochromatin and inversely regulate differentiation. Cell 152: 584–598. 23374351 10.1016/j.cell.2013.01.009

[WIJCHERSGR186643C68] Splinter E, de Wit E, Nora EP, Klous P, van de Werken HJ, Zhu Y, Kaaij LJ, van Ijcken W, Gribnau J, Heard E, 2011 The inactive X chromosome adopts a unique three-dimensional conformation that is dependent on Xist RNA. Genes Dev 25: 1371–1383. 21690198 10.1101/gad.633311 PMC3134081

[WIJCHERSGR186643C69] Splinter E, de Wit E, van de Werken HJ, Klous P, de Laat W. 2012 Determining long-range chromatin interactions for selected genomic sites using 4C-seq technology: from fixation to computation. Methods 58: 221–230. 22609568 10.1016/j.ymeth.2012.04.009

[WIJCHERSGR186643C70] Takizawa T, Gudla PR, Guo L, Lockett S, Misteli T. 2008 Allele-specific nuclear positioning of the monoallelically expressed astrocyte marker GFAP. Genes Dev 22: 489–498. 18281462 10.1101/gad.1634608 PMC2238670

[WIJCHERSGR186643C71] van de Werken HJ, de Vree PJ, Splinter E, Holwerda SJ, Klous P, de Wit E, de Laat W. 2012 4C technology: protocols and data analysis. Methods Enzymol 513: 89–112. 22929766 10.1016/B978-0-12-391938-0.00004-5

[WIJCHERSGR186643C72] Wakimoto BT, Hearn MG. 1990 The effects of chromosome rearrangements on the expression of heterochromatic genes in chromosome 2L of *Drosophila melanogaster*. Genetics 125: 141–154. 2111264 10.1093/genetics/125.1.141 PMC1203996

[WIJCHERSGR186643C73] Weddington N, Stuy A, Hiratani I, Ryba T, Yokochi T, Gilbert DM. 2008 ReplicationDomain: a visualization tool and comparative database for genome-wide replication timing data. BMC Bioinformatics 9: 530. 19077204 10.1186/1471-2105-9-530 PMC2636809

[WIJCHERSGR186643C74] Weierich C, Brero A, Stein S, von Hase J, Cremer C, Cremer T, Solovei I. 2003 Three-dimensional arrangements of centromeres and telomeres in nuclei of human and murine lymphocytes. Chromosome Res 11: 485–502. 12971724 10.1023/a:1025016828544

[WIJCHERSGR186643C75] Weiler KS, Wakimoto BT. 1995 Heterochromatin and gene expression in *Drosophila*. Annu Rev Genet 29: 577–605. 8825487 10.1146/annurev.ge.29.120195.003045

[WIJCHERSGR186643C76] Wiblin AE, Cui W, Clark AJ, Bickmore WA. 2005 Distinctive nuclear organisation of centromeres and regions involved in pluripotency in human embryonic stem cells. J Cell Sci 118: 3861–3868. 16105879 10.1242/jcs.02500

[WIJCHERSGR186643C77] Wilson AA, Kwok LW, Hovav AH, Ohle SJ, Little FF, Fine A, Kotton DN. 2008 Sustained expression of α1-antitrypsin after transplantation of manipulated hematopoietic stem cells. Am J Respir Cell Mol Biol 39: 133–141. 18323534 10.1165/rcmb.2007-0133OC PMC2542452

[WIJCHERSGR186643C78] Yasuhara JC, Wakimoto BT. 2006 Oxymoron no more: the expanding world of heterochromatic genes. Trends Genet 22: 330–338. 16690158 10.1016/j.tig.2006.04.008

[WIJCHERSGR186643C79] Zhang Y, McCord RP, Ho YJ, Lajoie BR, Hildebrand DG, Simon AC, Becker MS, Alt FW, Dekker J. 2012 Spatial organization of the mouse genome and its role in recurrent chromosomal translocations. Cell 148: 908–921. 22341456 10.1016/j.cell.2012.02.002 PMC3320767

